# Wild type measles virus attenuation independent of type I IFN

**DOI:** 10.1186/1743-422X-5-22

**Published:** 2008-02-03

**Authors:** Johan Druelle, Caroline I Sellin, Diane Waku-Kouomou, Branka Horvat, Fabian T Wild

**Affiliations:** 1 Inserm, U758, Lyon, F-69365 France ; Ecole Normale Supérieure de Lyon, Lyon, F-69007 France ; IFR128 BioSciences Lyon-Gerland Lyon-Sud, Université de Lyon 1; 21 Avenue Tony Garnier, 69365 Lyon Cedex 07 – France; 2 Centre National de Référence pour la Rougeole, Lyon, France

## Abstract

**Background:**

Measles virus attenuation has been historically performed by adaptation to cell culture. The current dogma is that attenuated virus strains induce more type I IFN and are more resistant to IFN-induced protection than wild type (wt).

**Results:**

The adaptation of a measles virus isolate (G954-PBL) by 13 passages in Vero cells induced a strong attenuation of this strain in vivo. The adapted virus (G954-V13) differs from its parental strain by only 5 amino acids (4 in P/V/C and 1 in the M gene). While a vaccine strain, Edmonston Zagreb, could replicate equally well in various primate cells, both G954 strains exhibited restriction to the specific cell type used initially for their propagation. Surprisingly, we observed that both G954 strains induced type I IFN, the wt strain inducing even more than the attenuated ones, particularly in human plasmacytoid Dendritic Cells. Type I IFN-induced protection from the infection of both G954 strains depended on the cell type analyzed, being less efficient in the cells used to grow the viral strain.

**Conclusion:**

Thus, mutations in M and P/V/C proteins can critically affect MV pathogenicity, cellular tropism and lead to virus attenuation without interfering with the α/β IFN system.

## Background

Mass vaccination with live attenuated measles vaccines has greatly reduced the incidence of this disease and its associated pathologies. Most vaccine strains were established after numerous passages on various cell lines. During this period of adaptation, the virus genome mutated in order to replicate efficiently in cell culture and thus, the original viral phenotype has been modified by mechanisms which are still poorly understood. The mutations observed in the RNA genome may be responsible for the replication of the clinical virus in its new host cell at different levels: entry, transcription, translation or budding.

Measles virus (MV), one of the leading causes of infant death in developing countries, is a member of the Paramyxovirus family. Like other viruses of this family, the MV negative RNA genome is protected by the N protein. Its association with the replicative complex (P and L proteins) constitutes the nucleocapsid. H (haemagglutinin) and F (fusion) proteins are surface glycoproteins, set in a lipid envelope, lined by the M (matrix) protein, and are responsible for the attachment and fusion processes. In addition to the structural proteins, the MV genome encodes for two accessory proteins, C and V [1].

For many years, few MV wild-type isolates were available for study. This was mainly due to the choice of the cell line used for virus isolation. Clinical or wild type MVs use CD150 (SLAM) as their main host cell receptor to attach to cells and so are most easily isolated on cell lines expressing this molecule [2]. The vaccine and vaccine-like strains, which readily multiply in cells lacking this receptor, were shown to be able to use an additional receptor, CD46, a ubiquitously expressed molecule [3,4]. Further, it was established that a critical amino acid (aa) in the MV H glycoprotein governed the use of the two receptors. Mutation of aa 481 from asparagine to tyrosine permitted the wild type strains to attach to CD46 [5]. *In vivo*, wild type viruses are reported to infect both endothelial and epithelial cells, which do not express CD150. Thus, it is not clear how the virus gets into the host cell. Two different hypotheses propose that either the wild type virus enters using CD46 as a low affinity receptor [6,7] or that there is another unidentified receptor involved [8-10].

Naniche et al. showed that in contrast to wild type MV strains, vaccine strains induced high levels of IFN in peripheral blood mononuclear cells (PBMCs) [11]. Moreover, the wild type strains were more sensitive to exogenous IFN. A number of studies have shown that MV, C and V accessory proteins may be implicated in both the inhibition of the induction and action of IFN [12-17]. However, it is still unknown whether the function of C and V proteins in the regulation of type I IFN system is altered after virus attenuation. Schlender et al. showed that a measles vaccine strain (Schwarz) replicates efficiently in plasmacytoid Dendritic Cells (pDCs, the major producers of type 1 IFN [18]) and blocks IFN induction by several ligands [19]. Nevertheless, the complexity and the diversity of the experimental systems previously used made a clear-cut interpretation of these data difficult in the estimation of the role of type I IFN in the attenuated MV phenotype.

It was shown that viruses isolated on B95a cells could induce in a monkey model all the clinical features observed in humans. However, adaptation of the virus to Vero cells attenuated the pathogenicity of the virus [20,21]. Sequence comparison of the 2 viruses showed that there were 5 aa changes in the polymerase (P/V/C and L) and 3 aa changes in the H [22] affecting the replication and transcription processes and also the syncytia formation. In a second study, the attenuating mutations were restricted to the P and M genes [23] with a deletion of the C gene. Recently, Tahara *et al*. adapted a wt strain to Vero cells and observed mutations in the M and L proteins. The adapted virus could grow in cells that did not express CD150 but was less efficient in the cell/cell fusion process [24]. The mutation E89K in M was then shown to be implied in alteration of the interaction between the M and H proteins [25].

In the present study, we compared G954-PBL, a MV wild type isolate propagated on PBMCs with G954-V13, a virus adapted from G954-PBL by 13 passages on Vero cells. Both strains were shown to have no differences in the H and F proteins and to use CD150 and not CD46 as a receptor [10]. Sequence analysis of both G954 viruses revealed that there were 5 mutations located only in the P/V/C and M genes. These mutations render the virus highly attenuated *in vivo*. Loss of pathogenicity could be related to different aspects of infection. Both G954 strains seemed to be restricted to specific cell types initially used to propagate the virus. Interestingly, the vaccine strain, Edmonston Zagreb (Ed-Zagreb), used as a control, was more robust than either of the G954 strains, multiplying in different cell types. Surprisingly, despite the differences in the P/V/C genes and the current belief that viral sensitivity to and induction of type I IFN correlate with an attenuated phenotype, this study shows the existence of exceptions to this dogma where virus attenuation is not linked to α/β IFN system.

## Methods

### Virus strains and cell lines

The wild-type MV strain, G954-PBL (genotype B3.2), was isolated in Gambia in 1993 and was propagated on activated human PBMCs. The Vero adapted strain, G954-V13 was obtained after 13 successive passages of a G954-PBL sample on Vero cells, [10]. MV vaccine Edmonston-Zagreb was kindly provided by D. Forcic and R. Mazuran (Immunology Institute of Zagreb, Croatia). Vesicular stomatitis virus (VSV) (Indiana strain) was propagated on Vero cells.

Measles viruses were titrated on Vero/CD150 cells by the standard plaque assay method as previously described [10]. For the establishment of viral kinetics, each time point was obtained individually. Infections were performed at a MOI of 0,1.

B95a, Vero, and Vero/CD150 cells were propagated in Dulbecco's modified Eagle's medium (Invitrogen) supplemented with 2 mM L-glutamine, 100 U of penicillin/ml, 0.1 mg of streptomycin/ml,10 mM HEPES, and 10% fetal calf serum or 2% for infections.

### Isolation and infection of human haematopoietic cells

Human PBMCs were prepared from whole blood of healthy donors (Etablissement Français du Sang, Lyon, France) by Ficoll-Hypaque density gradient centrifugation (Eurobio, France). pDCs were isolated by magnetic activated cell sorting (MACS) using the BDCA-4 dendritic cell isolation kit from Miltenyi Biotec. Prior to positive selection, monocytes, B cells, T cells, NK cells, red cells and macrophages were depleted by negative selection. Cells were incubated with an antibody cocktail directed against CD3, CD8, CD14, CD16, CD19, CD35, CD56 and glycophorin-A, then with Biomag Goat anti-mouse IgG magnetic beads (Quiagen) and finally separated using a Biomag magnet. PDCs were labeled with anti-BDCA-4 antibody coupled to colloidal paramagnetic micro beads and passed through a magnetic separation column (LS column; Miltenyi Biotec). The purity of isolated pDCs (BDCA2 positive, CD123-positive) was between 75% and 95%. pDCs from individual donors were used separately in all experiments and were not pooled. Contaminating cells were mainly monocytes. After isolation, cells were infected for 2 hours, then supernatants were removed and cells were cultivated in RPMI supplemented with 10% FCS at 37°C and 5% CO_2 _with 10 ng/mL of IL-3 for the pDC (10^5 ^cells/mL) and 1 μg/mL PHA, 50 U/mL IL-2 for the other cells (PBMCs, CD3^+^CD19^+^, monocytes ; 10^5 ^cells/mL).

### Infection of mice

Heterozygous one-week-old suckling CD150 transgenic mice [26], backcrossed or not in a type I IFN receptor deficient background [27] and their nontransgenic littermates were infected intranasally (i.n.) by application in both nares of 10 μl of MV (10^3 ^PFU). Clinical signs of disease and the weight of the mice were assessed daily for 8 weeks after infection. Mice were bred at the institute's animal facility (Plateau de Biologie Experimentale de la Souris, IFR128 BioSciences Lyon-Gerland, France), and *in vivo *protocols were certified by the Comité Rhone-Alpes d'Ethique pour l'Expérimentation Animale (CREEA).

### Determination of MV-specific antibodies in murine serum by ELISA

Sera were taken from G954-V13 infected mice at 60 days after infection from the retro-orbital vein or by intra-cardiac punction and tested for anti-MV antibodies by enzyme-linked immunosorbent assay (ELISA) as described previously [26]. The titer of N-specific antibodies in each serum sample was determined using a standard curve established with sera from mice immunized with MV in complete Freund's adjuvant and expressed in relative units.

### Extraction of MV-specific RNA

For quantitative PCR, total RNA was obtained directly from the supernatant of infected cells or control non-infected cells, using the Nucleospin RNA Virus kit (Macherey-Nagel, Düren, Germany), according to the manufacturer's protocol. For *in vivo *experiments, total RNA was extracted from murine brains and lungs at 10 days post infection with RNA-NOW (Biogentex, Ozyme, France) and treated with DNase I (Sigma).

### Detection and quantification of MV-specific RNA

Detection of efficient replication in mice brains (presence of mRNA coding for N), was performed as previously described [26]. For determination of viral genome production, cDNA was obtained using the Superscript II kit (Invitrogen) and further diluted to perform quantitative PCR using a Platinum SYBR Green qPCR super mix uracil DNA glycosylase kit (Invitrogen). The RT reaction was specific using the following primer (corresponding to the N region of the genome): 5'-GACATTGACACTGCATC-3'. The Quantitative PCR experiments were performed with this primer as forward and 5'-GATTCCTGCCATGGCTTGCAGCC-3' as reverse. QPCR was performed with an ABI Prism 7000 SDS, and results were analyzed using ABI Prism 7000 SDS software available from the Genetic Analysis Platform (IFR128 BioSciences Lyon-Gerland). In order to normalize the results, the ubiquitin housekeeping gene was quantified [26]. The level of expression of the gene of interest in an unknown sample was calculated from the real-time PCR efficiency of primers and the crossing point deviation of the unknown sample versus a standard, as described previously [28]. Briefly, these standard references were included in each PCR run for every analyzed gene in order to standardize the PCR run with respect to RNA integrity, sample loading, and inter-PCR variations. The calculated relative expression represents, therefore, the ratio of the expression level of gene of interest versus the expression level of the housekeeping gene. Otherwise, when the level of expression of none of the housekeeping genes tested was found to be stable, results were normalized in function of the initial number of cells.

### Nucleic acid sequencing

Experiments were performed as described in Kouomou et al [10]. Briefly, PCR products were electrophoresed on a 1.2% agarose gel, and then purified using a QIAquick Gel Extraction kit (Qiagen, Courtaboeuf, France) following the manufacturer's instructions. Purified PCR products were sequenced with the ABI prism Big Dye Terminator Cycle Sequencing Ready Reaction Kit (PE Biosystems, Langen, Germany). The reaction products were analyzed in an ABI Prism 3100 automatic sequencer (Perkin Elmer, Langen, Germany). The MV G954-PBL and G954-V13 sequences were deposited in Genebank under the accession numbers: EF565854 (N gene), EF565855 (P gene, G954-PBL), EF565857 (M gene, G954-PBL), EF565859 (L gene), EF565856 (P gene, G954-V13) and EF565858 (M gene, G954-V13).

### IFN-α/β detection assay

UV-inactivated cell culture supernatants were serially diluted (2-fold) and added to confluent Vero monolayer cells. After incubation for 24 h at 37°C, the cells were infected with VSV at 0.1 PFU/cell. Cytopathic effects were determined after fixation with formalin and methylene blue coloration 24 h later. Titration end-point represented dilutions that gave VSV-induced lysis of 50% of the cells. IFN titers are expressed as International Units per milliliter with reference to a standard IFN curve obtained using α-IFN (Sigma).

## Results

### Adaptation of wild type MV to Vero cells induced 5 mutations in the P/V/C and M genes

In a previous study [10], we isolated MV (G954-PBL) from the lymphocytes of a patient and maintained the isolate either in PHA-activated human PBMCs or adapted the virus to Vero cells. During this adaptation to Vero cells, we reported no changes in the amino acid sequences of the two viral glycoproteins, H and F [10]. Although the Vero infected cells expressed large amounts of the two glycoproteins at the cell surface, no fusion (syncytia) was observed. However, infection of Vero cells expressing the MV receptor CD150 readily induced fusion [10]. Three additional passages on Vero cells did not modify this viral phenotype. In order to identify the mutations implicated in the adaptation of the virus (G954-V13) to Vero cells, we sequenced the complete genomes of both viruses. There were a total of 5 nucleotide changes which led to coding changes. These are shown in table 1 (P, E242V ; V, H232D ; C, F93S and V130A ; M, E89K).

**Table 1:** Summary of nucleotide and deduced amino acid differences between the G954-PBL and G954-V13 strains.

Nucleotide				Amino Acid			
Gene	Position	G954 PBL	G954 V13	Protein	Position	G954 PBL	G954 V13

P/V/C	2106	T	C	C	93	Phe	Ser
	2217	T	C	C	130	Val	Ala
	2499	C	G	V	232	His	Asp
	2531	A	T	P	242	Glu	Val
							
M	3702	G	A	M	89	Glu	Lys

### The Vero-adapted strain, G954-V13, is highly attenuated in vivo

Intranasal infection of CD150 transgenic suckling mice with the G954-PBL strain leads to MV spread to different organs and to the development of a lethal neurological syndrome [26]. To study the pathogenicity of the G954-V13 Vero-adapted virus, transgenic CD150 suckling mice were inoculated intranasally with either the wild type G954-PBL or the adapted G954-V13 virus (table 2). Whereas the G954-PBL infected mice died within 15 days post infection (pi), no deaths were observed for the G954-V13 infected mice during the period of observation (90 days). Ten days after infection, when a high level of G954-PBL replication is observed [26], some of the infected mice were sacrificed and the presence of virus in the different organs was studied by RT-PCR. In the case of the G954-PBL infected mice, the distribution of the virus was similar to that previously described [26]. In the G954-V13 infected animals, MV was not detected by the technique used. However, at 60 days pi, the mice exhibited anti MV-N antibodies in their sera as detected by ELISA. Infection with UV-inactivated virus did not induce antibody production, strongly suggesting that generation of antibodies requires initial replication of G954-V13 after intranasal infection of CD150 transgenic mice. Therefore, G954-V13 replicates in this transgenic model without provoking any pathological effect demonstrating that MV adaptation to Vero cells is associated with an important loss of viral pathogenicity *in vivo*.

**Table 2:** Pathogenicity of G954 MV strains in vivo

Mice genotype (no. of mice)	Viral strain	Mortality rate (time/days)	MV replication (10 days pi) (*)		anti-N response (15 days pi)(†)
			brain	lung	

C57/Bl6 (8)	G954 PBL	0 %	-	-	-
	G954 V13	0 %	-	-	-
CD150 tg (6–8)	G954 PBL	100% (9–15 d)	+++	+	++
	G954 V13	0%	-	-	+/++
	UV inactivated G954 V13	0%	nd	nd	-
CD150/IFNARKO (8–10)	G954 PBL	100% (9–11 d)	nd	nd	nd
	G954 V13	0%	nd	nd	+/++

(*)determined by RT PCR on N mRNA: +++ > 10× housekeeping gene expression; + > 0,1× housekeeping gene expression ; – beyond limit of detection ; nd not determined

(†) determined by ELISA on N specific sera antibodies: ++ between 1 and 10 arbitrary units; + between 0,1 and 1 arbitrary units; – beyond 0,1 arbitrary units

Four of the 5 mutations differing G954-PBL and G954-V13, are located in the P/V and C genes. These proteins are known to interfere with the production and signaling of type I IFN, suggesting potential importance of type I IFN in G954-V13 attenuation. Therefore, we studied the pathogenicity of G954 strains in CD150 transgenic mice crossed into a type I IFN receptor KO background. While intranasal G954 V13 infection was again not lethal for transgenic mice, the infection with G954-PBL resulted in death of all the animals within 11 days (lethal outcome between day 9 and day 11). The absence of pathogenicity of G954-V13 in mice lacking type I IFN receptor strongly suggested that G954 V13 attenuation could be independent of type I IFN.

### Adaptation of MV restricts its replication to specific cell types

Although the transgenic murine model is a convenient system to test different aspects of MV infection, it cannot reflect completely the physiopathology in humans. Therefore, we further analyzed the properties of G954 viruses in different primate cell types.

The attenuated phenotype of G954-V13 could reflect its ability to replicate in different tissues. Moreover, the E89K mutation in the M protein of another wt strain of MV permitted an efficient replication in Vero cells while limiting the cell/cell fusion process [25]. In order to verify if such a phenomenon was observed with our strains, we compared the replication of both G954 viruses in several primate cell types and used the Edmonston Zagreb strain as a vaccine reference.

**Figure 1:**
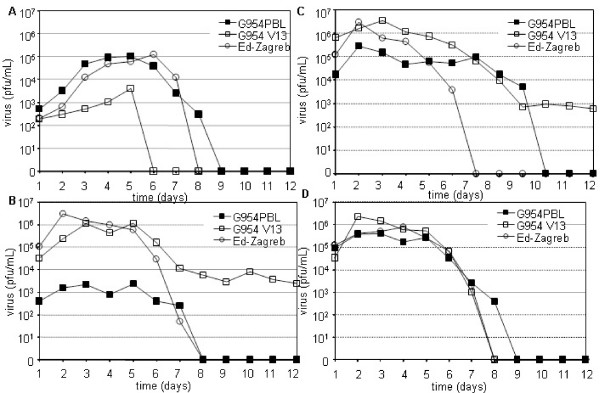
**Adaptation to a specific cell type limits replication of MV**. Replication kinetics of MVs in PBMCs (A), in Vero cells (B), in Vero/CD150 cells (C), in B95a cells (D). For each experiment, 10^5 ^cells were infected at a MOI of 0,1. Each time point consists of the mean of 2 independent experiments. Vero/CD150 cells were used for the titration.

PBMCs from healthy donors were infected with either G954-PBL or G954-V13 or Ed-Zagreb (MOI = 0.1) and the production of virus monitored daily (figure 1A). G954-PBL readily infected these cells with a peak of virus production on day 4. In contrast, G954-V13 virus poorly replicated: 100 fold less in these cultures than its parental strain. The Ed-Zagreb vaccine strain replicated almost as well as the wt strain.

The G954-V13 strain grew well in Vero cells (figure 1B), multiplying far more efficiently than G954-PBL. However, we did not observe any difference in syncytia formation between G954-PBL and G954-V13. The expression of CD150 on Vero cells did not modify the kinetics of G954-V13 replication. Similar results were obtained with the Ed-Zagreb vaccine strain. In the case of the wt strain, the availability of CD150 enhanced by almost 100 the yield of infectious virus (figure 1C).

All three viruses efficiently replicated in B95a cells, which express CD150 but not CD46. G954 V13 yield was 10 times greater than the wt and Ed-Zagreb infections the first 2 days of infection (figure 1D). Thus, the restriction of G954 strains to specific cell types did not seem to rely on known receptor expression.

At day 3 or 4 pi, i.e. when the virus yields were the highest, we performed RT-QPCR analysis on infected cultures. The number of MV genomes present in those cultures is shown in table 3. G954-V13 and Ed-Zagreb infections of PBMCs were 20 times less productive than infections of Vero/CD150 cells, while there were 10 fold more genomes of G954-PBL in infected PBMCs than in the Vero/CD150 cells.

**Table 3:** Number of copies of MV genome in different infected cell types

	PBMCs	Vero/CD150	pDCs
G954 PBL	3,3.10^5 ^*	3,8.10^4^	8.10^3^
G954 V13	1,6.10^5^	3,2.10^6^	2.7.10^3^
Ed-Zagreb	1,6.10^5^	3.2.10^6^	8.10^3^
G954 PBL UV	< 10	< 10	< 10
G954 V13 UV	< 10	< 10	< 10

* Data represent the number of copies of MV genomes deduced from RT-QPCR results obtained from infected cultures extractions when maximum PFU were released (8.104 cells infected at 0,1 PFU/cell).

Thus, viral adaptation to a specific cell type could be linked to a more efficient production of infectious particles and a greater accumulation/production of genomes in cell culture and not necessarily to restrictions at the entry level.

### Sensitivity of MV G954-PBL and G954-V13 to IFN

Previous studies showed that wild type MV strains are more sensitive than vaccine strains to type I IFN in PBMCs [11] and that V and C proteins can interfere with type I IFN signaling [13-16]. Therefore, we studied the sensitivity of G954 and Ed-Zagreb strains to type I IFN. As PBMCs are a very heterogeneous cell population, Vero/CD150 cells were also included in the study. These cells have the added advantage of not synthesizing IFN while being sensitive to its protective effect which means that any effect can be correlated to exogenously added IFN. Moreover, we studied the kinetics of infection prior to or following addition of type I IFN. This enabled us to study both the inhibition of virus production as well as delay in the establishment and duration of infection.

Addition of different amounts of type I IFN to Vero/CD150 cells prior to infection revealed that G954 viruses were inhibited to similar levels. Treatment of cells 48 hours prior to infection with 100 IU of type I IFN reduced infectious virus production by both viruses by approximately 80–90 % and was completely inhibited with 500 IU when assayed 3 days after infection (table 4). Pre-treatment of the cells with type I IFN for shorter periods revealed similar profiles except that higher concentrations of IFN were required to inhibit viral infection, suggesting a threshold effect. Finally, although the Ed-Zagreb infection was more resistant to a pretreatment of 8 and 24 hours than both G954 viruses, it showed similar resistance at the 48 h point.

**Table 4:** Inhibitory efficacy of a type I IFN pre-treatment on Vero/CD150 cells before MV infection

		Concentration of IFN added (IU/mL)				
Time before infection	Strain	5000	1000	500	100	0

8 h	G954-PBL	100*	92	95	63	0
	G954-V13	100	95	89	56	0
	Ed-Zagreb	100	79	65	58	0
24 h	G954-PBL	100	100	100	95	0
	G954-V13	100	100	100	67	0
	Ed-Zagreb	100	100	100	43	0
48 h	G954-PBL	100	100	100	91	0
	G954-V13	100	100	100	80	0
	Ed-Zagreb	100	100	100	81	0

* Inhibition indices were calculated as follow: 100 × [1 – (PFU(type I IFN = x)/PFU(type I IFN = 0)]

**Figure 2:**
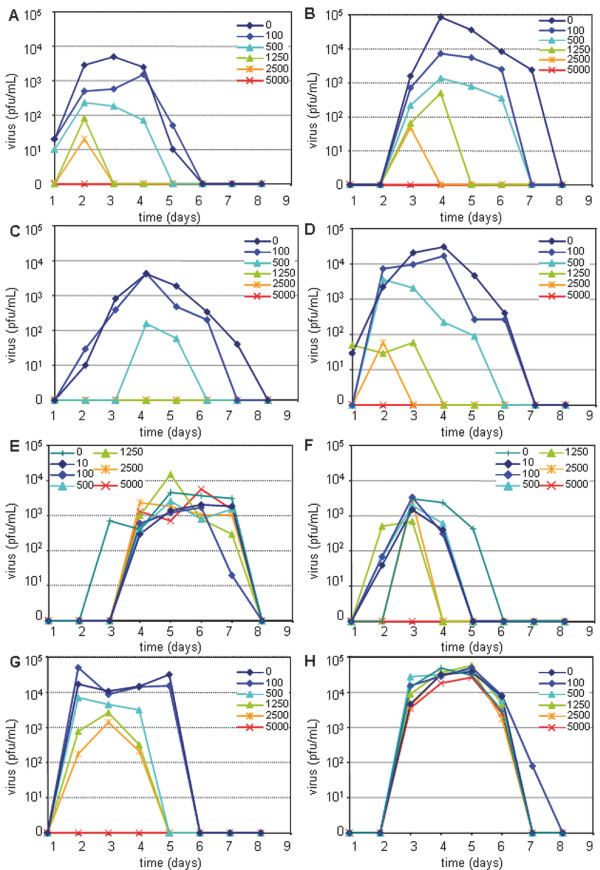
**Viral resistance to type I IFN induced protection depends upon the cell type used for viral adaptation**. G954-PBL infections of Vero/CD150 cells could be blocked with high doses of type IFN but the efficacy relied on the delay before treatment [(A): 2 hours (C): 12 hours between infection and type I IFN addition]. Infections with G954-V13 were less affected by type I IFN [(B): 2 hours (D): 12 hours between infection and type I IFN addition]. Infection of PBMCs by wt MV was not affected by type I IFN regardless of dose [(E): 2 hours between infection and type I IFN addition]. However, high doses of type I IFN could inhibit the G954-V13 strain infection [(F): 2 hours between infection and type I IFN addition]. Ed-Zagreb infections of both Vero/CD150 cells [(G): 4 hours between infection and type I IFN addition] and PBMCs [(H): 2 hours between infection and type I IFN addition] were relatively unaffected by type I IFN. Cells were infected at a MOI of 0,1 during 2 hours then washed. Various dilutions of type I IFN were added to cell cultures at specified times.

We next examined the effect of type I IFN during the MV infection. Different quantities of IFN were added to Vero/CD150 cells infected with G954-PBL, G954-V13 or Ed-Zagreb (MOI = 0,1) at 2, 4 or 12 hr post infection (figure 2A–D, G). The later the IFN was added the less effect it had on the inhibition of infectious virus production. Treatment with more than 1250 IU/mL of IFN 2 h pi blocked the infection by the wt strain. The infection was delayed and produced less infectious virus in proportion to the quantity of IFN added. IFN added 12 h after infection did not slow down the production of infectious MV. In the case of G954-V13, the effect of type I IFN was much less important. The infection was never delayed, slightly hampered and shortened, proportionally to the added dose. Interestingly, the infection by Ed-Zagreb was far more robust. Independently of the delay between type I IFN treatment and infection, there was a slight dose effect (figure 2G and unpublished results): the vaccine strain infection of Vero/CD150 cells was less affected by type I IFN. Thus, it appears that type I IFN-induced protection of Vero/CD150 cells was less effective in the case of infection by the Vero adapted strain, G954-V13. This type of resistance could be linked with a better adaptation of MV to the Vero cell environment.

When the protective effect of type I IFN on MV infection of PBMCs was studied, the wt strain was not inhibited despite the addition of high quantities of IFN (up to 5000 IU/mL). On the contrary, infection by G954-V13 strain was delayed, shortened and of lesser amplitude, proportionally to the IFN concentration and totally blocked with 5000 IU/mL of type I IFN. Finally, there was no effect of time between infection and IFN treatment on the virus replication in PBMCs for both analyzed viruses (figure 2E–F and data not shown). Infection of PBMCs by the Ed-Zagreb virus was unaffected by the different conditions of type I IFN tested (figure 2H). Thus, G954 strains seemed to be rather resistant to type I IFN since the protective effects occurred only when infections were performed on cells less permissive to a specific strain. However, Ed-Zagreb exhibited a strong resistance to type I IFN, independently of the cell type tested.

### Induction of type I IFN by MV infection

**Figure 3:**
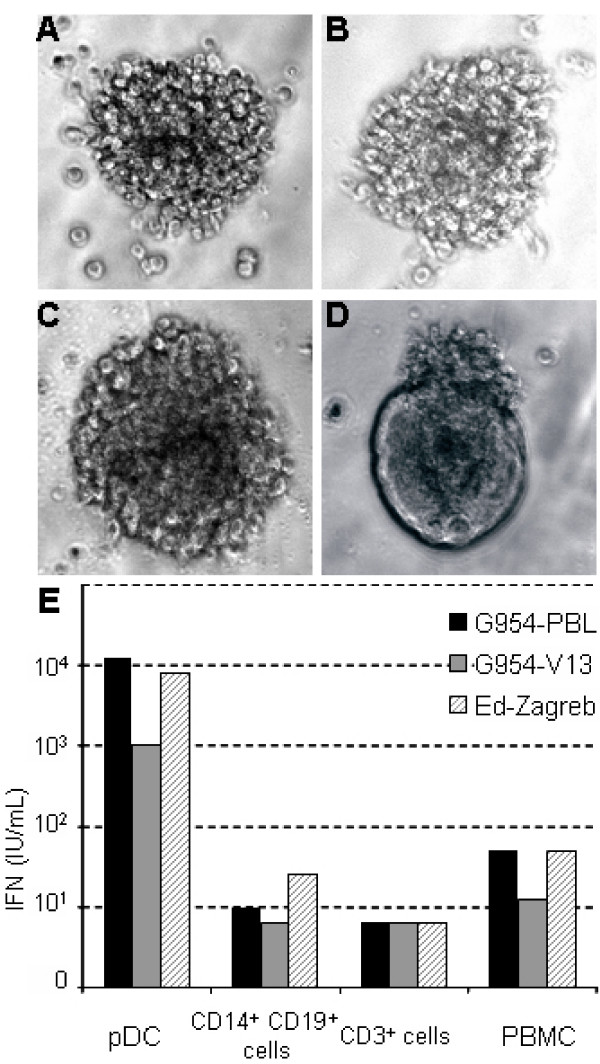
**Attenuation of G954 strain is not linked to type IIFN induction**. (A-D): Cytopathic effects of MV infection on pDCs. Cells were infected at a MOI of 0,1. Photographs were taken when the maximum of cytopathic effects was observed (2–4 days pi). (A): non infected pDCs; (B): Infection by G954 PBL; (C): G954 V13; (D): Edmonston Zagreb strain induces syncytia formation. Magnification ×400. (E): pDCs are the main producers of type I IFN following MV infection. Haematopoietic cells were infected at a MOI of 0,1 with G954 and Ed-Zagreb viruses. Type I IFN amounts were determined by biological assays on UV-inactivated supernatants harvested 3 days pi.

Plasmacytoid Dendritic Cells (pDCs) are the main producers of type I IFN in blood and lymph nodes. Unstimulated pDCs express CD46 at the cell surface, but not CD150 [29]. Schlender et al have shown that pDCs are infectable by the Edmonston-Schwarz vaccine strain of MV but do not produce IFN during the first 36 hours after infection [19]. To study the permissivity of these cells to different MV, pDC cultures were infected for 3 days with G954-PBL, G954-V13 or Ed-Zagreb. Neither G954 virus induced syncytia formation in the cultures nor were any infectious virus particles detectable, whereas infection with the Ed-Zagreb strain induced cell fusion without infectious virus production (figure 3A–D ). Quantitative RT-PCR studies on the infected cells showed that RNA replication/transcription could occur in pDCs (table 3). The pDCs infected with the wt strain and Ed-Zagreb contained 3 fold more genomes than those infected with G954-V13. The presence of MV genomes was not detected after infection with UV-treated virus (table 3) confirming the active replication of MV in pDCs.

To study the induction of type I IFN by these viruses, PBMCs and fractionated preparations (pDCs, CD14+ CD19+, and CD3+ cells) were infected and the production of type I IFN measured 3 days later (figure 3E). The infected pDCs had up to 1,000 fold higher quantities of α/β IFN than the other cells examined. The wild type G954-PBL virus induced 10-fold higher type I IFN amounts than the G954-V13 virus in pDCs and equal amounts as Ed-Zagreb. In all tested cell types, each MV strain induced production of type I IFN, although G954-V13 infection induced lower level, particularly in PBMCs and pDCs. Altogether, those results demonstrate that the G954-V13 attenuation/adaptation was not linked to an enhanced production of type I IFN by either of primary humans haemotopoietic cells analyzed in this study.

## Discussion

We have adapted a wild type MV to Vero cells and shown that the adapted strain and the parent strain differ from each other by only 5 coding mutations. Although the differences we have observed were located in the P/V/C and M genes, they were different from mutations observed in previous studies [21-23,30] where viruses were attenuated by passaging on Vero cells and then tested in a monkey model. Our MV adapted to Vero cells (G954-V13) was strongly attenuated when inoculated into CD150 transgenic mice. The E89K mutation in M protein has also been shown to be present in another Vero adapted strain [24] and was shown to permit the wt strain to replicate in Vero cells while provoking limited cell/cell fusion of CD150^+ ^cells. Our results support the proposed importance of the M E89K mutation in replication in Vero cells although we observed a better cytopathic effect in Vero/CD150 cells rather than defects in syncytia formation [data not shown and [10]]. A recent study shows that this mutation could affect MV growth by modifying the interaction between M and the cytoplasmic tail of the H protein [25]. Since the predicted domains on H for CD46 and CD150 binding are close, one could hypothesise that a stronger interaction of M with H could change the conformation of H and thus change the affinity of the CD46 binding site (R. Buckland, personal communication).

Parks et al. sequenced a number of the vaccine strains derived from the Edmonston isolate and identified amino acids shared by these attenuated viruses [31]. Eight amino acid coding changes were common to all vaccine strains and an additional two were conserved in all except the Edmonston Zagreb strain. They concluded that modulation of transcription and replication plays an important role in attenuation. Among the mutations found in G954-V13, only M E89K corresponds to an amino acid change observed in the transition toward vaccine strains in this study. The observation that the Edmonston Zagreb strain could readily replicate in PBMCs and Vero cells while resisting to type I IFN induced protection suggests the robustness of vaccine strains. Furthermore, it questions the notion of attenuated and vaccine strains since a virus which does not induce a pathology in humans could still exhibit strong deleterious effects in human cell cultures, demonstrating discrepancies of the virus pathogenicity *in vitro *and *in vivo*. Therefore, these observations beg the question of whether a vaccine phenotype can be predicted and engineered at the genetic level by using only *in vitro *approach.

Innate immunity is an important early response to viral infection. The accessory proteins of Paramyxoviruses, C and V, have been shown to be implicated in the suppression of this response, both in the induction and signalling of type I IFN [12-17,32]. Although in some of those studies, laboratory strains were poor inducers of type I IFN [14], other studies reported that vaccine strains induced 10 to 80 times more type I IFN than wt strains after infection of peripheral blood lymphocytes [11,33]. In contrast, in our study, the wild type and attenuated G954-V13 viruses as well as vaccine strain Ed-Zagreb induced similar quantities of type I IFN in these cells. We showed that following in vitro infection, the major cell population producing type I IFN was the pDCs for both the wild type and the attenuated strain. The inhibition of type I IFN production induced by vaccine MV strains observed in another study [19] was probably due to the shorter observation period (36 hours) than in our study (72 hours). Nevertheless, we cannot exclude potential interference of MV infection with TLR-induced type I IFN production by pDCs. Furthermore, it may also be possible that G954 forms part of a particular group of wt MV, able of good induction of type I IFN and then, during its attenuation, this property is preserved. Even if better induction and higher sensitivity to type I IFN is an attractive explanation for the mechanism of viral attenuation, this study strongly suggests that it is possible to achieve attenuation without perturbing interactions with the innate immune mechanisms.

Our results show that P/V/C mutations are not necessarily linked to modifications in type I IFN resistance and suggest rather that they have a role in the replicative process during infection. This is in agreement with previous studies on the negative effect of V and C proteins on transcription and replication [34-36]. The absence of the V protein was reported to delay replication [37] and the virus was less pathogenic in vivo [38,39]. The absence of the C protein reduced the virus yield both in vitro and in vivo [40]. Even the M protein has been shown to inhibit the replication process [41]. Recent studies showed that the P protein is involved in STAT1 phosphorylation [42] and thus can affect type I IFN efficacy. In our case such a role for P could not be observed. Moreover, other studies demonstrated that the adaptation of MV to Vero cells could induce differences in the amounts of viral proteins produced [43]. More quantitative experiments should be performed to assess if such a phenomenon is important in the adaptation of the G954 viral strain. Therefore, it may be very likely that differences between G954 strains are linked to P/V/C and/or M proteins via cell specific restrictions of viral replication, transcription and translation processes.

## Conclusion

The present study shows that adaptation of wild type MV to Vero cells induces a strong attenuation *in vivo*, which is independent of type I IFN. Identifying the exact role of each of the 5 mutations will determine their role in pathogenicity and could be performed by developing a recombinant virus strategy. Further analysis of the mechanisms implicated in the complex process of virus attenuation should pave the way towards developing new vaccines with a high capacity to induce specific host immune responses.

## Competing interests

The author(s) declare that they have no competing interests.

## Authors' contributions

JD participated in the conception of the study and performed the majority of the experiments and wrote the manuscript. CIS carried out ELISA assays on mice sera, participated in the *in vivo *assays and helped to draft the manuscript. DW carried out the nucleic acid sequencing and sequence alignment. BH helped in the design of the study, specially the in vivo assays and critically helped to draft the manuscript. TFW in the conception of the study, its design and coordination and helped to draft the manuscript. All authors read and approved the final manuscript.
